# Clinical features and metabolic reprogramming of atherosclerotic lesions in patients with chronic thromboembolic pulmonary hypertension

**DOI:** 10.3389/fcvm.2022.1023282

**Published:** 2022-11-15

**Authors:** Jixiang Liu, Ziyi Chang, Zhu Zhang, Bei Wang, Wanmu Xie, Qian Gao, Shuai Zhang, Yunxia Zhang, Han Tian, Zhihui Fu, Yishan Li, Kaiyuan Zhen, Shuangshuang Ma, Dingrong Zhong, Peiran Yang, Zhenguo Zhai

**Affiliations:** ^1^Department of Pulmonary and Critical Care Medicine, Center of Respiratory Medicine, China-Japan Friendship Hospital, Beijing, China; ^2^Peking Union Medical College, Chinese Academy of Medical Sciences, Beijing, China; ^3^National Center for Respiratory Medicine, Beijing, China; ^4^Institute of Respiratory Medicine, Chinese Academy of Medical Sciences, Beijing, China; ^5^National Clinical Research Center for Respiratory Diseases, Beijing, China; ^6^Department of Pathology, China-Japan Friendship Hospital, Beijing, China; ^7^The First Clinical Medical College, Shanxi Medical University, Taiyuan, China; ^8^State Key Laboratory of Medical Molecular Biology, Department of Physiology, Institute of Basic Medical Sciences, Chinese Academy of Medical Sciences and School of Basic Medicine, Peking Union Medical College, Beijing, China

**Keywords:** chronic thromboembolic pulmonary hypertension (CTEPH), pulmonary embolism, atherosclerosis, pulmonary endarterectomy (PEA), metabolic reprogramming

## Abstract

**Background:**

Chronic thromboembolic pulmonary hypertension (CTEPH) patients may present with atherosclerotic lesions in their pulmonary arteries, but their clinical characteristics remain unclear. The metabolic pathways associated with the atherosclerotic lesions may explain their occurrence and have implications for interventions, but they have not been investigated.

**Methods:**

We collected pulmonary endarterectomy (PEA) samples of CTEPH patients from December 2016 to August 2021. Following a detailed pathological examination of the PEA specimen, the patients were divided into those with and without lesions, and age- and sex matching were performed subsequently using propensity score matching (*n* = 25 each). Metabolomic profiling was used to investigate the metabolites of the proximal lesions in the PEA specimens.

**Results:**

In our study population, 27.2% of all PEA specimens were found to contain atherosclerotic lesions. CTEPH patients with atherosclerotic lesions were more likely to have a history of symptomatic embolism and had a longer timespan between embolism and surgery, whereas the classic risk factors of systemic and coronary circulation could not distinguish CTEPH patients with or without atherosclerotic lesions. Metabolomic profiling revealed that the formation of atherosclerotic lesions in CTEPH was closely related to altered glycine, serine, and threonine metabolic axes, possibly involved in cellular senescence, energy metabolism, and a proinflammatory microenvironment.

**Conclusion:**

The occurrence of atherosclerotic lesions in the pulmonary arteries of CTEPH was associated with symptomatic thromboembolic history and prolonged disease duration. The results revealed a new link between atherosclerotic lesions and aberrant amino acid metabolism in the context of CTEPH for the first time. This study has characterized the clinical and metabolic profiles of this distinct group of CTEPH patients, providing new insights into disease pathogenesis and potential interventions.

## Introduction

Chronic thromboembolic pulmonary hypertension (CTEPH) is a progressive pulmonary vascular disease caused by single or recurrent pulmonary thromboembolism, leading to elevated pulmonary vascular resistance and maladaptive right ventricular failure (1–3). Removal of organized thrombotic materials and neointima by pulmonary endarterectomy (PEA) is the gold standard therapy for operable CTEPH (4), conferring a 3-year survival rate of up to 89% in operated patients (5). Despite significant improvement in hemodynamic parameters and cardiac function, owing to the frequent occurrence of residual pulmonary hypertension (PH), combined implementation of medical therapy and/or intervention may become necessary after the PEA operation (6).

The characteristics of major vascular remodeling can be revealed by studying tissues removed by PEA. Varied pathological features have been reported, probably reflecting the different stages in the development of CTEPH (7, 8). In general, four major pathological alterations are observed within the specimens from PEA, including neointima formation, atherosclerotic lesions, thrombosis, and recanalization (7). Macrophage-derived foam cells and cholesterol clefts are found in the atherosclerotic lesions in CTEPH (7, 9). However, it remains uncertain whether the traditional cardiovascular risk factors and the thromboembolus-derived atherosclerotic lesions in CTEPH are clinically related (10, 11).

Atherosclerotic lesions do not develop in pulmonary arteries under the normal environment of low pressure and high flow, but they may form during the development of PH (9, 12). Atherosclerotic lesions were found in approximately 30% of CTEPH cases (7, 13). Arbustini et al. reported that atherosclerotic plaques with glycophorin-rich pultaceous cores were more common in CTEPH than those in plexogenic PH (14). Nevertheless, the predisposing factors and clinical relevance of these atherosclerotic lesions in CTEPH have rarely been discussed.

It has been speculated that the atherosclerotic lesion formation in CTEPH may be associated with local metabolic changes (15). To address this question, we have adopted a metabolomic approach to examine the metabolite alterations and define the endophenotypes of the disease. This study aimed to investigate the clinical characteristics of CTEPH patients with atherosclerotic lesions and further explore the relevant metabolic pathways associated with atherosclerosis in the pulmonary artery.

## Materials and methods

### Study population

Patients who underwent PEA with deep hypothermic circulatory arrest were consecutively included at the China-Japan Friendship Hospital from December 2016 to August 2021. The pre-operative assessment for PEA was conducted by a multidisciplinary team that included surgeons, interventional radiologists experienced in pulmonary vascular imaging, and pulmonologists with expertise in PH. During the study period, no technical or medical management changes occurred. Patients with pulmonary artery sarcoma (PAS) or preoperative mean pulmonary arterial pressure (mPAP) <25 mmHg were excluded. CTEPH was defined as previously described (16). The project was approved by the Ethics Committee of the China-Japan Friendship Hospital, and written informed consent was obtained from each participant.

### Data collection

All data were collected from the medical records using standardized data collection forms. The age of the lesion was calculated as the timespan between the first episode of acute pulmonary embolism and the PEA in years (7). For patients with a clear history of thromboembolism, the age of the lesion started from the determination of PE symptoms. For those patients with CTEPH and asymptomatic thromboembolic events, we defined the onset of symptom as the first episode of the disease, such as dyspnea, chest pain, syncope, and hemoptysis. Persistent PH was defined by an mPAP ≥30 mmHg obtained from right heart catheterization 2–3 days after operation in the intensive care unit, as described previously (17, 18).

### Tissue collection and histopathological procedures

Proximal lesions dissected from the main or lobar pulmonary arteries were photographed and then fixed in 4% paraformaldehyde overnight. Cross-sections were cut with a thickness of 5 μm for hematoxylin–eosin (H&E) staining. Atherosclerotic lesions were characterized by the accumulation of both foam cells and cholesterol crystals (7, 14). All specimens were independently reviewed by two experienced pathologists under light microscopes to determine for the presence of atherosclerotic lesions.

Meanwhile, to confirm the deposition of lipid, Oil-Red-O staining was performed. Briefly, PEA tissue was cut into 8-μm-thick sections with a cryostat microtome and fixed with 10% buffered formalin before being submerged in Oil-Red-O working solution for 20 min. Tissue sections were then rinsed and counterstained with hematoxylin and mounted in glycerine jelly. To assess the abundance of macrophages in atherosclerotic lesions, immunohistochemistry labeling of the macrophage marker CD68 was performed as described previously (6).

### Metabolomic profiling

The atherosclerotic lesions of the pulmonary arteries were clearly identified, and metabolomic analysis was conducted using tissues adjacent to the area of H&E staining. Non-targeted metabolomics were derived from samples of six atherosclerotic lesions and five non-atherosclerotic lesions of pulmonary arteries. Briefly, 800 μl of cold methanol was added to 30 mg of PEA tissue to precipitate the metabolites. The supernatant was dried and then resuspended in 200 μl of methanol. The metabolites were detected by a liquid chromatograph-mass spectrometer (LC-MS) platform (Waters, UPLC; Thermo, Q Exactive). Positive ion mode was used for the analysis of the difference in metabolites. Multivariate statistical analysis was performed using SIMCA-P Software (Umetrics AB, Umea, Sweden), including the principal component analysis and orthogonal projections to latent structures-discriminate analysis (OPLS-DA). The difference in metabolites used in this study was screened by a *t*-test *P*-value < 0.05 and variable weight of the OPLS-DA model >1. In addition, commercial databases including the Kyoto Encyclopedia of Genes and Genomes (KEGG)^1^ and MetaboAnalyst^2^ were utilized to search for the pathways of the metabolites (19).

### Statistical analysis

Propensity score matching was applied to control confounding factors such as selection bias. Continuous variables characterized by a normal distribution were expressed as mean and standard deviation (SD). Parameters without a normal distribution were expressed as the median and interquartile range (IQR). Continuous variables were compared using Student’s *t*-test or Mann–Whitney U test based on their distribution. Categorical variables were presented as frequencies and percentages and compared using the Chi-square test or Fisher’s exact test, where necessary. The risk factor of atherosclerosis was evaluated through univariate logistic regression models. A *P*-value < 0.05 was considered to be statistically significant. All analyses were carried out using SPSS (version 21.0, IBM Corp.).

## Results

### Characteristics of the study population

**FIGURE 1:**
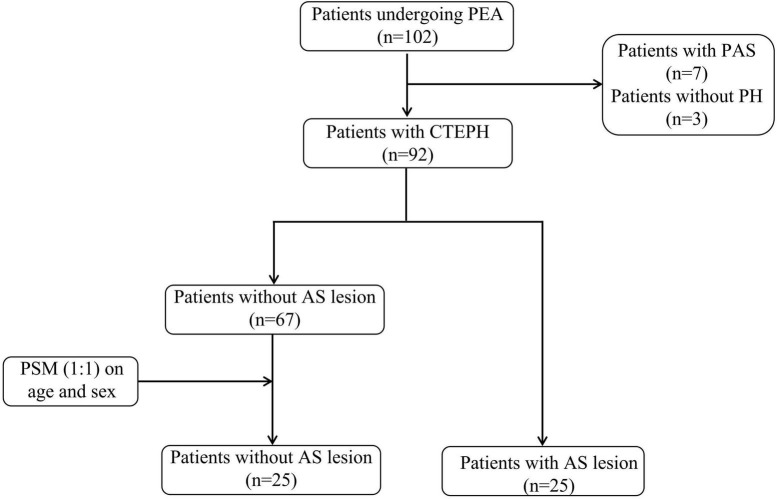
The flowchart of the patient selection process. PEA, pulmonary endarterectomy; PAS, pulmonary artery sarcoma; PH, pulmonary hypertension; CTEPH, chronic thromboembolic pulmonary hypertension; AS, atherosclerosis; PSM, propensity score matching.

As shown in the flowchart in Figure 1, a total of 102 patients underwent PEA surgery from December 2016 to August 2021. After excluding 7 patients diagnosed with PAS, 95 patients were included in this study, including 3 patients without PH. The characteristics of all patients with chronic thromboembolic pulmonary disease are shown in Supplementary Table 1. Of note, no evidence of an atherosclerotic lesion was found in the three excluded patients with chronic thromboembolic pulmonary disease who did not meet the hemodynamic criteria for PH. Atherosclerotic lesions were found in 25 (27.2%) of all patients with CTEPH (the atherosclerotic lesion group) and were not found in the proximal lesions of the pulmonary artery in the remaining 67 patients. There was a tentative difference in age among the two groups (56 vs. 51 years, *P* = 0.094), although it was not statistically significant. Since the development of atherosclerosis is known to be significantly associated with age and gender, propensity score matching was performed to reduce bias (10). In this way, 25 patients without atherosclerotic lesions (the non-atherosclerotic lesion group) were selected in the subsequent analysis.

The demographic characteristics and hemodynamic features of the two patient groups are shown in Table 1. Patients with atherosclerotic lesions were more likely to have a history of symptomatic embolism (88 vs. 56%, *P* = 0.012), including pulmonary embolism and deep vein thrombosis. However, there were no significant differences between the two groups of patients in co-morbidities and hemodynamic parameters.

**TABLE 1:** Demographic and hemodynamic characteristics of the study population.

	Atherosclerotic lesion (*n* = 25)	Non-atherosclerotic lesion (*n* = 25)	*P*-value
Age, years	56.0 (47.0–61.0)	54.0 (46.5–62.5)	0.808
Male, *n* (%)	15 (60)	15 (60)	1.000
Acute PE/DVT, *n* (%)	22 (88)	14 (56)	0.012
Thrombophilia*, *n* (%)	2 (8)	2 (8)	1.000
**Preoperative hemodynamics**			
mRVP, mmHg	30.0 (23.0–34.0)	27.0 (23.0–31.0)	0.299
mPAP, mmHg	45.0 (38.0–51.0)	45.0 (39.0–53.5)	0.141
>45 mmHg, *n* (%)	12 (48)	11 (44)	0.777
PVR, Wood units	11.50 (8.59–17.29)	11.54 (9.43–12.86)	0.961
>12.5 Wood units, *n* (%)	12 (48)	9 (36)	0.390
**WHO functional class**			
III/IV, *n* (%)	19 (76)	17 (68)	0.529
**Echocardiography**			
RV, mm	50.84 ± 8.36	49.92 ± 7.62	0.892
TAPSE, mm	15.37 ± 2.88	14.56 ± 3.29	0.557
S′, cm/s	9.34 ± 2.05	9.10 ± 2.55	0.632
PA, mm	34.80 ± 7.58	33.92 ± 6.51	0.944
NT-proBNP, pg/ml	1399 (681–2625)	799 (257.5–3427)	0.299
SvO_2_, %	67.0 (58.0–73.85)	68.0 (56.0–73.0)	0.832
Preoperative PH treatment, *n* (%)	11 (44)	8 (32)	0.387
Post-operative mPAP, mmHg	24.0 (20.5–28.0)	26.0 (21.5–34.0)	0.339
Persistent PH^#^, *n* (%)	6 (24)	9 (36)	0.355

DVT, deep vein thrombosis; PE, pulmonary embolism; mPAP, mean pulmonary artery pressure; mRVP, mean right ventricular pressure; PVR, pulmonary vascular resistance; WHO, World Health Organization; RV, diameter of right ventricle (basal); TAPSE, tricuspid annular plane systolic excursion; S′, tricuspid systolic velocity; PA, diameter of pulmonary artery; NT-proBNP, N-terminal pro B-type natriuretic peptide; SvO2, mixed venous oxygen saturation; PH, pulmonary hypertension.

*Thrombophilia included patients with protein C deficiency, protein S deficiency or antithrombin deficiency. ^#^Persistent PH, was defined by an mPAP ≥30 mmHg 2–3 days after pulmonary endarterectomy.

**FIGURE 2:**
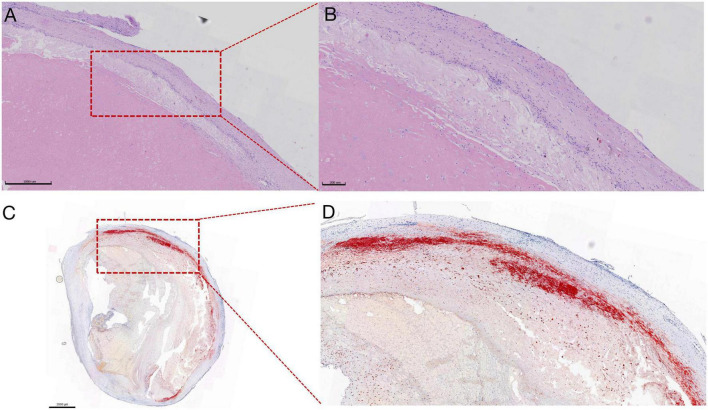
Representative images of atherosclerotic lesions in PEA specimens revealed by H&E and Oil-Red-O staining. **(A)** Irregular-shaped cholesterol crystallization and the cluster of foam cells derived from macrophages engulfing the lipid, scale bars: 1,000 μm. **(B)** Magnification of the picture on the left. **(C)** The bright red area shows apparent lipid deposition, localized inside the neointima, and adjacent to the thrombotic materials, scale bars: 2,000 μm. **(D)** Magnification of the picture on the left.

**FIGURE 3:**
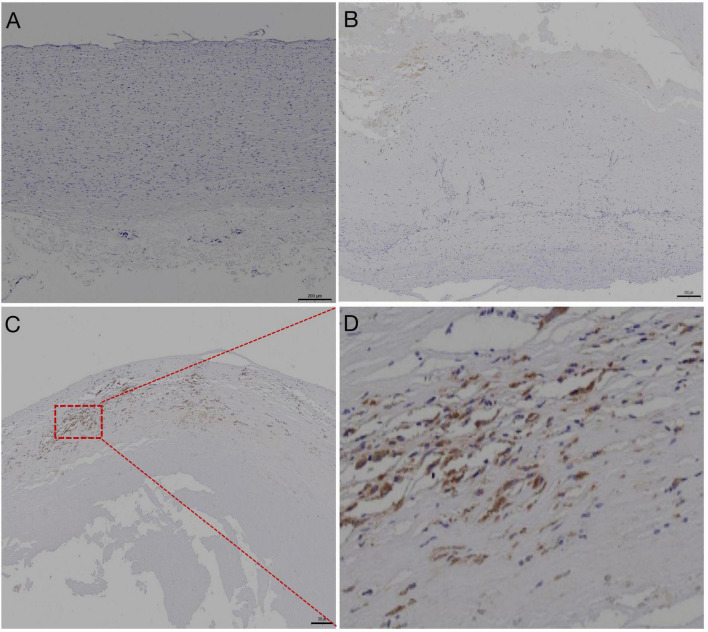
Representative images of pulmonary artery immunolabeled with anti-CD68 antibodies. **(A)** Negative immunohistochemical staining of the pulmonary artery from healthy lung transplant donor, scale bars: 200 μm. **(B)** Relatively lower abundance of CD68+ macrophages in the non-atherosclerotic lesion of CTEPH, scale bars: 200 μm. **(C)** Strong positive immunoreactivity of CD68+ macrophages was found in the atherosclerotic lesion, outlined by the red box, of the pulmonary artery resected by pulmonary endarterectomy. Scale bar: 200 μm. **(D)** Magnified view of panel **(C)**.

The atherosclerotic lesions of CTEPH were characterized by the accumulation of lipid-laden foam cells derived mainly from macrophages, as well as cholesterol crystals displaying as clefts (Figures 2A,B). Oil-Red-O staining showed that typical lipid depositions were mainly localized inside the neointima and adjacent to the thrombotic materials (Figures 2C,D). Furthermore, calcification was observed in the PEA tissues by H&E staining from 40% (10/25) of CTEPH patients with atherosclerotic lesions, higher than 8% (2/25) of those without an atherosclerotic lesion. There was no macrophage infiltration in the pulmonary artery from the healthy lung transplant donor (Figure 3A). As reflected by CD68 immunolabeling, macrophage infiltration was clearly found in the atherosclerotic lesion in PEA-derived tissue. Meanwhile, we have also found moderate macrophage accumulation in the non-atherosclerotic area of the CTEPH pulmonary artery (Figures 3B–D).

### Risk factors of atherosclerotic lesions and association with severity of chronic thromboembolic pulmonary hypertension

To identify the risk factors for atherosclerotic lesion in CTEPH, various clinical parameters were compared between the two groups of patients. No apparent differences were found between the two groups of patients, as shown in Table 2. Based on these results, the formation of atherosclerotic lesions in the pulmonary artery might not be associated with the traditional risk factors for atherosclerosis, such as increased plasma cholesterol, smoking history, and diabetes. The higher incidence of symptomatic thromboembolic history in the CTEPH patients with atherosclerotic lesions suggested that the occurrence of atherosclerotic lesions might be related to the direct endothelial damage caused by the presence and compression of embolus. Moreover, the age of the lesion of patients with atherosclerosis was longer than that of patients without atherosclerosis (8.0 ± 6.5 vs. 4.7 ± 4.2 years, *P* = 0.039), as shown in Table 2. Therefore, it was possible that the formation of unresolved clots caused long-term damage to the endothelium, leading to atherosclerosis. In this way, the atherosclerotic lesions probably developed secondary to the accumulation of thromboembolic materials and were related to long-term injury of the endothelium due to the thromboembolus.

**TABLE 2:** Risk factors of atherosclerosis in CTEPH with and without atherosclerotic lesion.

	Atherosclerotic lesion (*n* = 25)	Non-atherosclerotic lesion (*n* = 25)	*P*-value
BMI, kg/m^2^	25.05 ± 4.26	24.00 ± 3.21	0.335
Smoking history, *n* (%)	9 (36)	11 (44)	0.564
Diabetes, *n* (%)	1 (4)	0	1.000
COPD, *n* (%)	3 (12)	1 (4)	0.609
Coronary artery disease, *n* (%)	6 (24)	5 (20)	0.733
Age of the lesion*, years	8.0 ± 6.5	4.7 ± 4.2	0.039
**Serum lipids**			
Total triglyceride, mmol/L	1.18 (0.60–1.57)	1.05 (0.59–2.22)	0.960
Total cholesterol mmol/L	3.66 (2.84–4.54)	3.49 (2.76–5.13)	0.904
HDL, mmol/L	0.96 (0.80–1.11)	0.96 (0.68–1.06)	0.536
LDL, mmol/L	2.20 (1.67–2.89)	2.02 (1.67–3.37)	0.920
Homocysteine, umol/L	13.9 (6.9–19.3)	16.1 (8.3–21.9)	0.472
SBP, mmHg	115 (109–124.5)	118 (106.5–135.75)	0.284
DBP, mmHg	78 (71–84.5)	81 (72.25–89.5)	0.423

BMI, body mass index; COPD, chronic obstructive pulmonary disease; HDL, high-density lipoprotein; LDL, low-density lipoprotein; SBP, systolic blood pressure; DBP, diastolic blood pressure.

*The age of the lesion was defined as the timespan (years) between the first episode of pulmonary embolism and the PEA surgery.

No significant difference was found in preoperative hemodynamics and right ventricular function between the two groups. Not surprisingly, a significant decrease in mPAP after surgery was confirmed in all CTEPH patients (Table 1). There was no significant difference in persistent PH between CTEPH patients with and without atherosclerotic lesions (24 vs. 36%, *P* = 0.355). At this early time point, there was no correlation between atherosclerotic lesions and postoperative persistent PH.

### Differential metabolites of atherosclerotic lesions in chronic thromboembolic pulmonary hypertension

**FIGURE 4:**
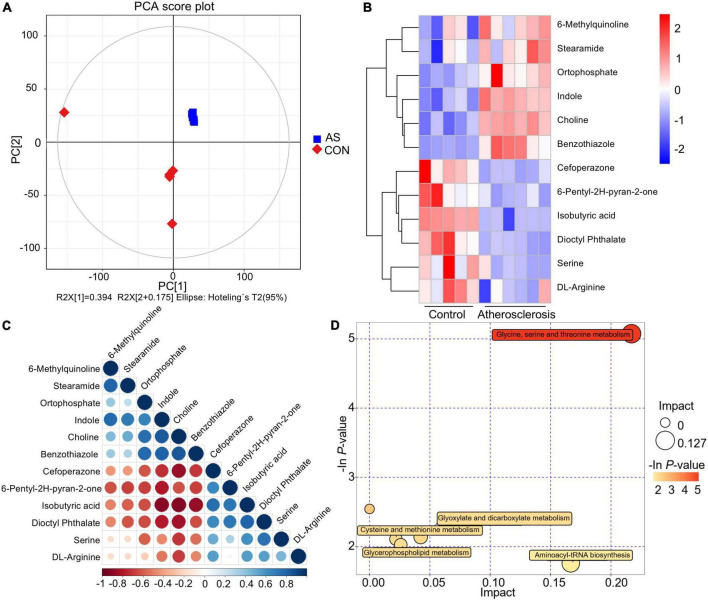
Differential metabolites and key metabolic pathways of atherosclerotic lesion. **(A)** Principal component analysis score plots. **(B)** Heatmap of the differential metabolites. **(C)** Associations between the differential metabolites. **(D)** Bubble plot of pathway enrichment analysis.

To assess the metabolites involved in the formation of atherosclerotic lesions in CTEPH, non-targeted metabolomics were performed on the pulmonary artery tissues from PEA. Baseline data of the randomly selected patients are shown in Supplementary Table 2. Significant differences in metabolites between the two groups were suggested by principal component analysis score plots (Figure 4A). The heatmap of the major differential metabolites is shown in Figure 4B. Several key metabolites were significantly elevated in the atherosclerotic group compared with the non-atherosclerotic patients, including indole and choline. On the other hand, several metabolites were decreased in the atherosclerotic group, including isobutyric acid and serine. Figure 4C reflects the association between these differential metabolites. The pathway enrichment analysis further revealed that the differences in glycine, serine, and threonine-related metabolism were related with the atherosclerotic lesions (Figure 4D). In brief, altered glycine, serine, and threonine metabolism may play a potential role in the occurrence of atherosclerotic lesions in CTEPH pulmonary arteries.

## Discussion

In the present study, we described the clinical characteristics of CTEPH patients with atherosclerotic lesions and discovered the underlying metabolic alterations of these lesions. Our data indicated that atherosclerotic lesions in CTEPH were likely to be associated with a history of symptomatic embolism and a prolonged timespan between thromboembolism and PEA surgery, instead of the common risk factors of atherosclerosis in the systemic and coronary circulation. In line with these clinical findings, the formation of atherosclerotic lesion was related to aberrant amino acid metabolism, possibly responsible for cellular senescence and abnormal energy metabolism. The clinicopathological description of atherosclerosis and its associated changes in metabolites revealed by this study contribute to our understanding of an important subgroup of CTEPH and provide novel insight into the pathogenesis of this pulmonary vascular disease.

The proportion of atherosclerotic lesions in patients with CTEPH population was 27.2% in our patient population, which is consistent with previous pathological studies (7, 13). In our study, patients with atherosclerotic lesions were more likely to have had a history of symptomatic embolism. Patients with a prolonged timespan between thromboembolism and PEA surgery had a significantly higher incidence of atherosclerotic lesions, possibly due to persistent damage of the pulmonary artery by the fibrotic clots over time. However, in terms of this timespan, as nearly a quarter of the population did not have a history of thromboembolic events, and given that the initial episode of thromboembolism may not even be the first thromboembolic event in patients with a history of thromboembolism, the age of the lesion may be undetermined or underestimated for all patients in this study. As for the implication of atherosclerosis of the outcome of surgical treatment, Corsico et al. have demonstrated that after the perioperative period, the early hemodynamic benefits of PEA remain largely stable over 5 years (20). We therefore concluded that at an early time point, patients with and without atherosclerotic lesion had no difference in risk of persistent or residual PH. A possible reason for this might be that atherosclerotic lesions were accompanied by organized fibrotic materials that could be removed more completely by surgery. Taken together, we have characterized this unique subset of CTEPH patients in detail, revealing the clinical features of patients with CTEPH and an atherosclerotic lesion in the pulmonary artery.

Atherosclerosis is mainly characterized by an accumulation of cholesterol crystals and foam cells derived from macrophages, under the influence of a variety of predisposing factors (21, 22). However, the common risk factors of atherosclerosis in the systemic and coronary circulation, such as smoking, altered blood lipid, and pulmonary emphysema, were not found in CTEPH patients with atherosclerotic lesions in the present study. It is well known that the major pulmonary artery and its branches are rarely affected by atherosclerotic lesions due to their relatively low blood pressure and high flow (12). In contrast, during the development of PH, atherosclerotic lesions may occur in the main pulmonary artery (9, 12, 23). Atherosclerotic lesions in the pulmonary vascular bed have also been noted in pulmonary arterial hypertension, revealed by autopsy, possibly as a result of shear stress-induced endothelial injury and subsequent inflammation (24, 25). During the histopathological examination in our study, typical lipid deposition was found to be located under the thrombi and was suspected to be a secondary lesion caused by chronic fibrotic clots, which is consistent with previous reported findings (26). We also found that atherosclerotic lesions were mostly localized in the proximal region, while angiogenesis was found in the distal region (results not shown), suggesting that atherosclerotic lesions may cause defective angiogenesis in some way, such as a local release of pro-inflammatory cytokines, which deserves further investigation. It has been proposed that “multiple hits” contribute to the pathogenesis of CTEPH following thromboembolism, including local release of inflammatory factors, endothelial dysfunction, and macrophage polarization (27, 28). Intriguingly, the same factors are also known to induce the formation of atherosclerotic lesions. Collectively, this manifestation of atherosclerotic lesions following chronic thromboembolism is distinct from systemic and coronary atherosclerosis but may instead be attributable to a unique local microenvironment that is influenced by a variety of stimuli.

In agreement with this notion of an altered microenvironment, an infiltration of macrophages was found in the atherosclerotic lesions, suggesting that foam cells in the atherosclerotic lesion could be derived from activated macrophages. Consistently, Quarck et al. found that the accumulation of inflammatory cells, represented by macrophages, was found in atherosclerotic and thrombotic lesions (7). The influence of pro-inflammatory mediators on pathophysiological processes such as macrophage polarization is a major underlying cause of many chronic inflammatory diseases (29, 30). Choline, a key metabolite that we identified in the atherosclerotic tissue in CTEPH, has been proven to be involved in the macrophage-foam cell formation and the development of atherosclerotic lesion (31). In addition, serine deprivation inhibits macrophage interleukin-1β (IL-1β) production to modulate macrophage function, resulting in less inflammation upon pro-inflammatory challenge (32). Of note, serine metabolism is necessary for glutathione synthesis to support IL-1β production (33). It has also been proven that the glycine-related metabolic axis is involved in energy metabolism (19, 34). More importantly, previous studies showed that alterations in amino acid metabolism could accelerate the development of coronary atherosclerosis and ischemic heart disease (35, 36). The differential metabolites in atherosclerotic lesions found in this study were significantly related to altered energy metabolism, which were consistent with metabolomic investigations of atherosclerosis using coronary tissues (37, 38). Therefore, we believe that the chronic thromboembolism has a certain impact on the proinflammatory cytokine production and altered energy metabolism of the local pulmonary artery, thereby affecting lipid deposition. The findings of this study may promote further research on the link between metabolic reprogramming, proinflammatory microenvironment, and atherosclerotic lesions in CTEPH. Further studies are required with targeted metabolomics, validation, and mechanistic exploration in order to understand the roles of specific metabolic pathways.

The current European Respiratory Society statements recommend screening patients with persistent or new-onset dyspnea after pulmonary embolism despite standard anticoagulation, indicating that earlier diagnosis and intervention may determine the outcome of the patient (2). In our study, the difference of the age of the lesion between the two groups represented the prolonged disease duration of patients with atherosclerotic lesions. Meanwhile, an atherosclerotic lesion was observed in none of the three chronic thromboembolic disease patients without PH. We thus presumed that the atherosclerotic lesions developed during the late stage of remodeling of the pulmonary arteries. Although the existing evidence does not support a relation between an atherosclerotic lesion and the severity of CTEPH based on preoperative hemodynamics and right heart size and function, the observed correlation with the course of disease still supports the notion of earlier screening patients with acute PE. It is possible that early effective intervention before or during the development of atherosclerotic lesions may arrest the progression of CTEPH.

We acknowledge the limitations of our study. First, this study was derived from a single-center retrospectively observational study and could be better supported with replications from independent centers. The absence of a follow-up assessment has limited us in deducing conclusions related to long-term prognosis. We also acknowledge the potential selection bias that may arise from propensity score matching. Additionally, a quantitative assessment of macrophage activation was not performed and will be explored in future mechanistic studies. However, the evidence of an atherosclerotic lesion in the pulmonary artery in CTEPH and relevant alterations of metabolites were the main focus and findings of the current study.

## Conclusion

In summary, we found that CTEPH patients with atherosclerotic lesions generally had a higher proportion of symptomatic embolism and prolonged disease duration. A potential linkage has been suggested between atherosclerotic lesions and an aberrant metabolic program, which has been suggested to play a key role in proinflammatory mediator production and energy metabolism. Collectively, we have characterized the clinical features and the metabolic landscape of this distinct group of CTEPH patients, providing novel insights into the pathophysiology and potential therapeutic strategies for the development of CTEPH.

## Data availability statement

The raw data supporting the conclusions of this article will be made available by the authors, without undue reservation.

## Ethics statement

The project was approved by the Ethics Committee of the China-Japan Friendship Hospital. The patients/participants provided their written informed consent to participate in this study.

## Author contributions

JL, ZheZ, and PY conceived the project, interpreted the data, and drafted the manuscript. ZC, BW, and DZ performed morphometric analysis. HT, YL, ZF, KZ, and SM collected patient information. WX, QG, SZ, YZ, and ZhuZ provided technical support and contributed to the discussion of the project. All authors critically reviewed the manuscript and made intellectual contributions to the study.

## Footnotes

1
http://www.genome.jp/kegg/

2
http://www.metaboanalyst.ca/
