# Supplementary material for: Clinical features and metabolic reprogramming of atherosclerotic lesions in patients with chronic thromboembolic pulmonary hypertension
Source: Front Cardiovasc Med. 2022 Nov 15;9:1023282. doi: 10.3389/fcvm.2022.1023282 (PMC9705335; doi:10.3389/fcvm.2022.1023282)
Supplement: Supplementary file 1 [file Data_Sheet_1.docx]

Table S1 Demographic and hemodynamic characteristics of the all study population

|  | Atherosclerotic lesion  (n=25) | Non-atherosclerotic lesion* (n=70) | *P* value |
| --- | --- | --- | --- |
| Age, years | 56.0 (47.0-61.0) | 51.0 (39.0-61.0) | 0.094 |
| Male, n (%) | 15 (60) | 48 (68.6) | 0.436 |
| Acute PE/DVT, n (%) | 22 (88) | 42 (60) | 0.025 |
| Age of lesion, years | 3.0 (2.0-7.5) | 3.0 (1.5-7.3) | 0.949 |
| mPAP, mmHg | 45.0 (38.0-51.0) | 45.0 (39.0-53.5) | 0.141 |
| PVR, Wood units | 11.50 (8.59-17.29) | 10.30 (6.57-13.24) | 0.347 |
| WHO functional class |  |  |  |
| III/IV, n (%) | 19 (76) | 49 (70) | 0.568 |
| Preoperative PH treatment, n (%) | 11 (44) | 22 (31.4) | 0.257 |

Abbreviations: DVT, deep vein thrombosis; PE, pulmonary embolism; mPAP, mean pulmonary artery pressure; PVR, pulmonary vascular resistance; WHO, world health organization; PH, pulmonary hypertension.

* The group of non-atherosclerotic lesion included 67 patients with CTEPH and 3 patients with chronic thromboembolic pulmonary disease without PH.

Table S2 Characteristics and hemodynamic features of patients of untargeted metabolomics

|  | Atherosclerotic lesion (n=6) | Non-atherosclerotic lesion (n=5) |
| --- | --- | --- |
| Age, years | 60.67±5.50 | 61.40±5.77 |
| BMI, kg/m^2^ | 23.14±1.29 | 23.68±3.28 |
| Male, n | 4 | 3 |
| History of acute PE/DVT, n | 5 | 4 |
| Thrombophilia*, n | 0 | 0 |
| Time from symptoms, years | 8.42±6.26 | 3.40±1.52 |
| Preoperative mPAP, mmHg | 54.17±20.09 | 49.40±8.74 |
| Postoperative mPAP, mmHg | 28.83±6.52 | 27.80±7.46 |
| PVR, Wood units | 17.32±5.99 | 15.22±5.48 |
| WHO functional class |  |  |
| III/IV, n | 5 | 3 |
| SvO_2_, % | 69.75±5.74 | 68.20±13.94 |
| Preoperative PH treatment, n | 3 | 1 |

Abbreviations: BMI, body mass index; DVT, deep vein thrombosis; PE, pulmonary embolism; mPAP, mean pulmonary artery pressure; PVR, pulmonary vascular resistance; WHO, world health organization;NT-proBNP, N-terminal pro B-type natriuretic peptide; SvO2, mixed venous oxygen saturation; PH, pulmonary hypertension.

*Thrombophilia included patients with protein C deficiency, protein S deficiency, antithrombin deficiency.
